# Macular Holes Secondary to Macular Telangiectasia Type 2: Pathogenesis, Clinical Features, Imaging and Surgical Management

**DOI:** 10.3390/jcm15197673

**Published:** 2026-10-03

**Authors:** Lihteh Wu, Genesis Chen-Li

**Affiliations:** Asociados de Macula, Vitreo y Retina de Costa Rica, Primer Piso Torre Mercedes Paseo Colón, San José 60612, Costa Rica

**Keywords:** macular hole, macular telangiectasia type 2, Müller cells, parafoveal telangiectasis, juxtafoveolar telangiectasia, internal limiting membrane flap

## Abstract

Full-thickness macular holes (FTMHs) are a rare but recognized complication of macular telangiectasia type 2 (MacTel2). Unlike idiopathic FTMHs, which are primarily driven by vitreofoveal traction, MacTel2-associated holes appear to arise predominantly from progressive neurodegenerative changes. Müller cell dysfunction, intraretinal cavitation, and outer retinal atrophy progressively compromise foveal architecture, ultimately predisposing to tissue dehiscence and full-thickness defects. Although vitreofoveal traction may contribute in individual cases, it is unlikely to be the principal mechanism in most affected eyes. Surgical treatment remains challenging, as current evidence is limited to case reports and small case series. Internal limiting membrane (ILM) flap techniques and, in selected cases, retinal graft or amniotic membrane transplant procedures have yielded encouraging anatomical closure rates, whereas conventional ILM peeling alone appears less predictable. Importantly, restoration of foveal continuity does not necessarily result in meaningful visual improvement, particularly in eyes with established photoreceptor and outer retinal damage. The durability of anatomical repair may be further compromised by continued disease progression. Persistent Müller cell dysfunction, intraretinal cavitation, and progressive outer retinal atrophy can undermine foveal support and lead to recurrent architectural disruption, including reopening of a previously closed hole. Although late recurrence has been documented, its incidence and determinants remain poorly defined because of the limited size and heterogeneity of the available literature. Visual prognosis appears to be determined largely by the amount of structurally and functionally viable retinal tissue remaining at the time of intervention. Smaller defects, less extensive cavitation, and preservation of the external limiting membrane and ellipsoid zone are associated with greater potential for visual recovery. In contrast, extensive cavitation, advanced outer retinal atrophy, and substantial Müller cell or photoreceptor loss are associated with limited functional improvement and may compromise long-term anatomical stability. Overall, a FTMH secondary to MacTel2 should be regarded as a distinct form of macular hole in which surgical repair occurs against a background of ongoing macular neurodegeneration. Preoperative evaluation of hole dimensions, foveal cavitation, and outer retinal integrity may assist in prognostication and in identifying eyes with the greatest likelihood of achieving durable anatomical closure and meaningful visual recovery.

## 1. Introduction

Macular telangiectasia type 2 (MacTel2) is considered to be a relatively rare condition with prevalence rates ranging from 0.0045% to 0.12%. However, these older population-based studies most likely underestimate its prevalence since their findings were based solely on fundus photographs [1,2]. The most recent population-based study used multimodal retinal imaging including fundus photos, optical coherence tomography (OCT) and fundus autofluorescence (FAF) to report an incidence of 0.7 per 100,000 persons per year in Olmsted County, Minnesota, USA [3]. MacTel2 is a genetically complex macular disorder with variable penetrance and expressivity that is characterized by progressive loss of Müller cells and photoreceptors accompanied by retinal vascular abnormalities [4]. An uncommon complication is a full-thickness macular hole (FTMH) [1,4]. Its prevalence has been estimated to be up to 5% [5,6,7,8]. A retrospective case series of 184 eyes from Türkiye with a minimum follow-up of three years, reported FTMH formation in 8 eyes. A small Japanese study of 16 eyes with MacTel2 that were followed for a mean of 8.2 years reported an eye with a FTMH at baseline and the development of a FTMH after 3 years of follow-up [6]. A retrospective multicenter cross-sectional study that included 129 patients from 6 tertiary hospitals from South Korea reported 4 (2.4%) eyes with a lamellar macular hole (LMH) and 5 (3%) eyes with a FTMH [7]. An OCT-based study of 332 eyes from India showed that the incidence of LMH was 54% and FTMH was 1% [8].

A macular hole (MH) is defined as a retinal defect in the macular area. The defect can be partial thickness, also known as an LMH, or an FTMH [9]. The reported global prevalence of MH varies widely from 0.2 per 1000 in the Blue Mountains Eye Study (Australia) to 3.3 per 1000 in the Baltimore Eye Survey (USA). The most recent study from Olmsted County in Minnesota, USA reports an age- and sex-adjusted incidence of 7.8 persons and 8.7 eyes per 100,000 population per year [10,11,12]. A FTMH can be classified according to its underlying pathophysiologic mechanism into primary or secondary MH. A primary MH arises from an abnormal vitreomacular traction [13,14,15]. In secondary MH, the MH develops as a direct or indirect result of an underlying ocular disease such as MacTel2 or injury.

There is currently an absence of consensus on what constitutes the best management for MH secondary to MacTel2. Since the pathologic mechanisms in this condition differ from those in primary MH, the anatomical closure and functional recovery after surgery may be different. The following narrative review aims to update the reader on the pathogenesis, current and future treatments of MH secondary to MacTel2.

## 2. Methods

We conducted a narrative review where the literature was identified through searches of PubMed, Embase, and Google Scholar, using a combination of the keywords “macular telangiectasia type 2” OR “MacTel type 2” OR “MacTel2” OR “idiopathic macular telangiectasia” OR “type 2 idiopathic macular telangiectasia” OR “parafoveal telangiectasia” OR “juxtafoveolar telangiectasis” AND “macular hole” OR “full-thickness macular hole” OR “lamellar macular hole” OR “foveal defect” OR “foveal dehiscence” from 1982 through September 1 2026. Examination of the reference lists of retrieved articles was assessed for additional relevant publications. All articles, regardless of language, that reported on the outcomes of MH secondary to MacTel2 were included. The initial search identified 588 articles. After removing 72 duplicates, 516 unique records were screened at the title and abstract level. Finally, 30 articles were included and underwent a full-text review. 

GPT-5.6 Luna (Open AI, San Francisco, CA, USA) was used to create Figure 1 and Figure 2.

## 3. Müller Cells

Müller cells are the principal glial cells of the vertebrate retina, spanning its full thickness from the internal limiting membrane (ILM) to the external limiting membrane (ELM) [16,17]. Through their extensive radial processes, they provide structural, metabolic, and homeostatic support to retinal neurons and interact with virtually all major retinal cell types, including neurons, astrocytes, microglia, endothelial cells, and pericytes. As the primary structural scaffold of the retina, Müller cells maintain retinal architecture through an extensive cytoskeletal network. They also form specialized junctions with neighboring neurons and contribute to ELM formation, thereby supporting photoreceptor organization and function [16,17].

Importantly, Müller cells exhibit marked topographic specialization across the retina, with particularly distinctive structural adaptations within the macula. Figure 1 illustrates the topographic specialization of Müller cells in the retina. The unique architecture of the fovea is associated with two specialized Müller cell populations that contribute to the organization and mechanical integrity of the central retina. In the foveola, Müller cells assume a characteristic inverted cone-shaped configuration, forming the structural scaffold that supports the foveal pit and preserves its highly specialized architecture [18,19]. More peripherally, within the parafovea, Müller cells adopt a distinctive Z-shaped arrangement, characterized by elongated processes that course obliquely through the Henle fiber layer toward the outer nuclear layer. By comparison, Müller cells in the peripheral retina exhibit a more conventional morphology, with relatively thicker, vertically oriented inner processes. These pronounced regional differences in Müller cell architecture underscore the specialized role of Müller glia in maintaining foveal structure and may help explain why disruption of Müller cell integrity has particularly important consequences in disorders characterized by selective macular degeneration, such as MacTel2 [18,19].

Müller cells within the macula possess a highly specialized metabolic phenotype that distinguishes them from their peripheral counterparts. In particular, macular Müller cells exhibit increased expression of phosphoglycerate dehydrogenase (PHGDH), the rate-limiting enzyme of the de novo serine biosynthetic pathway, together with greater glycolytic activity, enhanced mitochondrial function, and increased metabolic flux through serine biosynthesis. This metabolic specialization is likely adapted to the exceptional energetic and oxidative demands imposed by the macula, where high photoreceptor density and sustained metabolic activity necessitate tightly regulated substrate utilization and redox homeostasis. Because serine metabolism contributes critically to cellular redox regulation and sphingolipid homeostasis, perturbation of this metabolic network may render macular Müller cells particularly susceptible to dysfunction. Accordingly, impaired serine biosynthesis and its downstream metabolic consequences have emerged as a plausible mechanistic link between Müller cell metabolic dysfunction and the selective macular vulnerability characteristic of MacTel2 [20].

## 4. MacTel2

MacTel2 was initially described as an idiopathic juxtafoveolar retinal telangiectasis [21]. It is characterized by loss of retinal transparency in the macular area, right-angled venules, retinal crystalline deposits, retinal pigment plaques, LMH, lack of lipid exudation, foveal atrophy, and late subretinal neovascularization in some eyes. Rarely, a yellow foveal lesion or a FTMH may develop. In most cases, the structural changes begin in the temporal parafoveal area and spread circumferentially with disease progression [1,2]. All the structural alterations and functional abnormalities are limited to a horizontally oval-shaped area centered in the fovea measuring 3 × 2.5 mm, known as the MacTel area [22]. MacTel2 eyes exhibit a strong structure–function correlation between ellipsoid zone (EZ) loss on OCT and retinal sensitivity measured by microperimetry [23,24,25,26,27,28].

Serine and lipid dysregulation within the macula play a central role in the pathogenesis of MacTel2 [29]. As a precursor for sphingolipid synthesis and free radical scavenging, serine is vital to multiple metabolic pathways that maintain retinal homeostasis. Due to its high metabolic demand, the retina relies on both circulating serine and de novo serine synthesis [30,31]. Photoreceptors and other retinal neurons do not express phosphoglycerate dehydrogenase (PHGDH)—the rate-limiting enzyme in de novo serine biosynthesis. Consequently, the retinal pigment epithelium (RPE) and Müller cells serve as the primary producers of retinal serine [20,32,33,34,35].

Sphingolipids are crucial structural components of cellular membranes and act as key signaling molecules. Their synthesis depends on the condensation of fatty acyl-coenzyme A (CoA) with serine, a process mediated by serine palmitoyltransferase (SPT). Adequate serine availability is therefore critical to sustain normal sphingolipid metabolism, mitochondrial integrity, and overall cellular function. Under serine-deficient conditions, SPT utilizes alternative substrates such as alanine or glycine, resulting in the formation of cytotoxic 1-deoxysphingolipids (1-dSLs). At the cellular level, both reduced serine production and 1-dSL accumulation trigger mitochondrial disruption [36,37]. These atypical sphingolipids accumulate within the endoplasmic reticulum, Golgi apparatus, and mitochondria, where they activate the unfolded protein response in photoreceptors and Müller cells [38]. Furthermore, in vitro studies using human induced pluripotent stem cell (iPSC)-derived retinal organoids demonstrate that 1-dSL exposure directly induces photoreceptor apoptosis [38,39].

Patients with MacTel2 demonstrate a characteristic disturbance in serine metabolism, with reduced circulating serine and glycine concentrations and increased levels of deoxysphingolipids compared with healthy individuals [40,41,42]. Multimodal retinal imaging has further implicated Müller cells as an important cellular target of the disease [29,43,44], consistent with their apparent vulnerability to alterations in serine metabolism [1,29,43,44]. Experimental studies support a causal relationship between this metabolic disturbance and retinal dysfunction. Mice maintained on a serine- and glycine-deficient diet developed marked abnormalities in photopic and flicker electroretinographic responses, suggesting impaired retinal function under conditions of systemic serine depletion. Deoxysphingolipids, particularly 1-deoxysphingolipids (1-dSLs), have also been shown to induce photoreceptor death in retinal organoids [39]. Complementary ultrastructural connectomic analysis of an eye affected by MacTel2 demonstrated mitochondrial abnormalities extending throughout the retinal layers [45]. Moreover, in vitro suppression of de novo serine biosynthesis in Müller cells disrupted mitochondrial homeostasis and promoted cell death, with these effects becoming more pronounced under oxidative stress [35]. Studies of RPE cells differentiated from MacTel2 patient-derived iPSCs provide additional evidence of a persistent serine-deficient state within retinal cells [31]. Collectively, these findings suggest that impaired serine metabolism may contribute to MacTel2 through interconnected effects on Müller cell viability, mitochondrial function, photoreceptor integrity, and retinal homeostasis.

The US Food and Drug Administration (FDA) recently approved the ciliary neurotrophic factor implant revakinagene taroretcel-Lwey (Neurotech Pharmaceuticals Inc, Cumberland, Rhode Island, USA) in MacTel2 patients. Proprietary allogeneic RPE cells that have been transfected with the CNTF gene are encapsulated in a polymer implant with a semipermeable membrane that is sutured to the sclera at the pars plana. These cells are genetically engineered to continuously secrete CNTF at therapeutic doses at a steady rate into the vitreous cavity. The semipermeable membrane permits nutrients from the vitreous cavity to nourish the RPE cells and the CNTF to diffuse into the vitreous cavity but does not allow the cells of the host’s immune system to diffuse into the implant and attack the CNTF-producing RPE cells [46]. Two identically designed phase 3 trials recently demonstrated that treatment with revakinagene taroretcel-Lwey led to a 55% and 31% reduction in EZ area loss at 24 months of follow-up. Reported adverse events related to the CNTF implant included delayed dark adaptation and miosis. Surgical complications included a case of a device extrusion, but no cases of vitreous hemorrhage or endophthalmitis were reported [47,48]. Although CNTF therapy represents an important therapeutic advance, it does not address the underlying disease biology.

## 5. Macular Holes

MH repair is one of the success stories in the history of vitreoretinal surgery. From a condition that was deemed incurable, MH is currently one of the conditions that respond most favorably to vitreoretinal surgery. In 1869 Knapp [49] reported the first case of an MH in the literature in a patient who suffered eye trauma. A couple of years later, Noyes [50] provided the first clear, detailed ophthalmoscopic description of a traumatic MH. In 1900 Ogilvie [51] was the first to use the term “hole at the macula”. Soon thereafter alternative pathophysiologic mechanisms other than trauma were proposed. First, Kuhnt [52] and then Coats [53] suggested that not all MH were caused by trauma but could also be caused by a degenerative process. Zeeman [54] in 1912 and later Lister [55] in 1924 introduced the idea of vitreoretinal traction. Before modern vitreoretinal surgery, an MH was generally regarded as an irreversible condition for which no effective treatment existed. Diagnosis depended on clinical biomicroscopy, including contact lens examination and the Watzke–Allen test. More than half a century later, Gass [13,14,15] re-introduced the concept of vitreoretinal traction. Based on his biomicroscopic observations he hypothesized that contraction of the posterior vitreous cortex produced anteroposterior and tangential traction on the fovea, ultimately resulting in foveal dehiscence and MH formation. His staging system provided the conceptual framework for subsequent surgical intervention. His work suggested that vitreous traction might be surgically relieved before irreversible retinal dehiscence occurred [13,14,15]. The development of pars plana vitrectomy (PPV) by Machemer et al. [56] and subsequent improvements in vitreous surgery provided the technical foundation for testing this hypothesis. The modern era of MH surgery began with the pioneering work of Kelly and Wendel, [57] who demonstrated that PPV with removal of the posterior vitreous hyaloid, removal of any epiretinal membranes, gas tamponade with a non-expansile concentration, and postoperative face-down positioning could result in anatomical closure and visual improvement. Their initial 1991 report transformed the management of a condition previously considered essentially untreatable. In their original series, closure was achieved in approximately 58% of eyes [57]. By 1993, their larger series of 170 eyes demonstrated a 73% closure rate. Their early experience demonstrated that anatomical closure was possible and that successful closure could be accompanied by meaningful visual recovery [58]. Since then, the surgical techniques and instrumentation have evolved.

The introduction of OCT in the 1990s fundamentally changed both diagnosis and surgical decision-making [59]. Unlike biomicroscopy, OCT provided cross-sectional visualization of the fovea and vitreoretinal interface. OCT demonstrated that MH formation was not simply a consequence of tangential traction. Anteroposterior vitreous traction, vitreofoveal adhesion, intraretinal cystic changes, and progressive dehiscence could be visualized directly. The International Vitreomacular Traction Study Group subsequently developed an OCT-based classification system that defined FTMH as interruption of all retinal layers from the ILM to the retinal pigment epithelium, and incorporated hole diameter and vitreomacular traction into the classification [9]. This transition from clinical staging to quantitative OCT-based characterization was important because hole size became a major determinant of prognosis and surgical strategy.

The next major development was deliberate removal of the ILM. Although the original Kelly–Wendel description referred to removal of a “friable” membrane around the hole, subsequent investigators recognized that this structure likely included the ILM. In 1997, Eckardt and co-workers [60] formally described intentional ILM removal during macular hole surgery. The technique involved creating a controlled tear in the ILM and removing a circular area around the hole. Chromovitrectomy made ILM easier and safer [61]. ILM peeling leads to elimination of residual tangential traction; removal of a relatively rigid basement membrane surrounding the hole; reduction in the scaffold for subsequent epiretinal proliferation; increased retinal compliance around the fovea; and facilitation of centripetal movement of retinal tissue toward the hole. Thus, surgery evolved from simply relieving vitreous traction to actively modifying the mechanical properties of the foveal retina.

Comparative studies suggested substantially higher closure rates with ILM peeling. In one study, all 116 eyes undergoing ILM peeling remained closed compared with primary closure in 82% of eyes without peeling; reopening was also substantially more common in the non-peeling group [62]. The evidence was subsequently strengthened by randomized trials. A multicenter randomized trial of 141 patients found significantly greater early anatomical closure with ILM peeling (84% versus 48%) and substantially fewer reoperations. Although the primary visual acuity (VA) endpoint at 6 months did not differ significantly, the anatomical advantage established ILM peeling as the preferred technique for many stage 2 and 3 holes [63]. A Cochrane review of randomized trials similarly found that ILM peeling substantially increased the likelihood of primary and final hole closure and reduced the need for additional surgery, despite relatively limited evidence for a major long-term difference in VA [64]. Consequently, ILM peeling became a standard component of MH repair.

Despite the high success rates achieved with conventional ILM peeling, large MHs remain difficult to close. Holes greater than approximately 400 µm, chronic holes, highly myopic holes, and MH associated with retinal detachment or MacTel2 have traditionally had lower closure rates. In 2010, Michalewska and colleagues [65] introduced the inverted ILM flap technique for large MH. Instead of completely removing the ILM surrounding the hole, a peripheral ILM remnant was left attached and inverted over the hole. In their prospective randomized study of holes larger than 400 µm, they demonstrated closure in 98% of eyes treated with the inverted flap compared with 88% after conventional surgery. The inverted flap was also associated with fewer flat-open holes and better visual outcomes. The mechanism remains incompletely understood. Proposed mechanisms include provision of a scaffold for Müller cell proliferation; promotion of glial tissue bridging; stimulation of retinal tissue migration; facilitation of photoreceptor reorganization; and mechanical coverage of the foveal defect. The inverted flap therefore represented a conceptual transition from removing tissue to facilitate closure toward using residual tissue to actively promote closure. A meta-analysis involving 1403 eyes found that the inverted ILM flap surgery was associated with a higher overall closure rate than conventional ILM peeling, including in large idiopathic holes, myopic holes, and holes associated with retinal detachment [66].

The inverted flap has subsequently been modified into several configurations, including a complete inverted flap, a temporal inverted flap, pedicle ILM flap, free ILM flap transplantation, and multiple-flap techniques. These modifications attempt to retain the biological advantages of the ILM flap while reducing unnecessary manipulation of the perifoveal retina. This is particularly relevant because ILM peeling itself is not biologically neutral. Mechanical manipulation can produce inner retinal structural abnormalities, dissociated optic nerve fiber layer appearance, microscotomas, and potentially retinal sensitivity changes. Consequently, contemporary surgery increasingly considers how much ILM should be removed, rather than simply whether it should be removed. A randomized study comparing 2-disk-diameter and 4-disk-diameter ILM peeling found no significant overall difference in closure at 12 months, although larger peeling appeared advantageous in eyes with unfavorable macular hole configuration. This supports the broader concept of tailoring the extent of ILM manipulation to the anatomical characteristics of the hole.

Over the past decade, newer instrumentation and surgical techniques have allowed vitreoretinal surgeons to push the limits of MH repair. Amniotic membrane transplantation (AMT) and autologous retinal transplantation (ART) are placed over and within the MH respectively to bridge the tissue defect [67,68]. Others have used plasma rich in growth factors (PRGF) membrane and autologous blood as adjuvants [69,70].

## 6. Macular Holes Secondary to MacTel2

### 6.1. Pathophysiological Mechanisms

FTMH is a recognized, though not extremely common, complication of MacTel 2. In 1988 Patel and colleagues [71] reported a 37-year-old man who developed a LMH. This case most likely represents macular telangiectasia type 1 rather than MacTel2 [72]. The first case of a FTMH secondary to MacTel2 was reported by Olson and Mandava [73]. They suggested that MacTel2 somehow altered the relationship between the posterior hyaloid and the ILM thus promoting MH formation [73]. Koizumi and colleagues [74] described two patients with an LMH and an FTMH respectively. No obvious vitreous traction was observed in either eye. They proposed that Müller cell cone loss led to structural weakness in the central fovea, causing lateral separation of the photoreceptors, leading to MH formation. None of the above 3 cases underwent surgical intervention [73,74].

MH secondary to MacTel2 appears to develop predominantly through progressive Müller cell dysfunction and retinal tissue loss rather than the vitreofoveal traction that characterizes primary MH. MacTel2 is a metabolic neurodegenerative disorder associated with systemic serine deficiency, and Müller cells are particularly important to foveal integrity because they provide structural and trophic support while interacting closely with photoreceptors and the retinal vasculature. Disruption of Müller cell function may therefore compromise the structural stability of the fovea, initiating intraretinal cavitation and tissue loss, particularly within the inner and outer retinal layers temporal to the foveal center. Early OCT features, including intraretinal cavitations and ILM draping, suggest that this structural deterioration precedes the development of a FTMH. With disease progression, these cavitations may enlarge and coalesce, accompanied by photoreceptor degeneration, disruption of the EZ and ELM, and progressive outer retinal atrophy. Continued loss of retinal tissue may eventually culminate in foveal dehiscence and formation of an FTMH. Figure 2 illustrates the proposed pathophysiological model of an MH secondary to MacTel2. Thus, many MH secondary to MacTel2 appear to evolve from pre-existing lamellar defects or schisis-like cavitation rather than primary vitreomacular traction, although epiretinal membranes and subtle vitreofoveal traction may act as contributory factors in individual eyes [4,29,43,44,75].

Figure 3 illustrates a case where vitreofoveal traction contributes to the formation of an LMH. (A) Focal area of increased FAF in the temporal parafovea; (B) SD-OCT showing an inner retinal cavitation (white asterisk) with discontinuity of the ELM (red arrows) and EZ (white arrows) bands; (C) Focal area of increased FAF in the temporal parafovea; (D) SD-OCT showing vitreofoveal traction (white arrow) creating a LMH (asterisk).

### 6.2. Differentiation Between a Primary FTMH and FTMH Secondary to MacTel2

A FTMH secondary to MacTel2 exhibits morphological differences when compared to a primary FTMH. These variations stem entirely from their distinct pathophysiologic mechanisms. OCT is particularly helpful in differentiating one from the other. In addition, examination of the fellow eye is important since most cases of MacTel2 are bilateral [1,4]. In primary FTMH, the OCT is characterized by highly symmetric, steep, and edematous edges. The inner and outer retinal layers terminate sharply and vertically around the foveal core. There is a distinctive cuff of subretinal fluid surrounding the FTMH. In a FTMH secondary to MacTel2, cavitations may precede the formation of the FTMH. On OCT, the margins of the hole are asymmetric, sloping, and irregular. The foveal walls are frequently thinned or collapsed due to progressive tissue loss. Typically, there is no cuff of subretinal fluid surrounding the FTMH. Additionally, the hole itself is often subtly displaced temporally from the geometric center of the fovea, reflecting the classic temporal-perifoveal predilection of MacTel2. Figure 4 illustrates these differences. (A) SD-OCT of a primary FTMH; (B) SD-OCT of a FTMH secondary to MacTel2. Note the disrupted EZ (white arrows) and ELM (asterisks) on the temporal side of the MH. The inner retina is also thinner on the temporal side. The red asterisk denotes the FTMH.

### 6.3. Anatomic Outcomes of FTMH Secondary to MacTel2

Most eyes with an FTMH develop acute visual loss. However, a handful of cases have been reported to close spontaneously [76,77,78,79]. In one case, the MH was small, measuring 207 µm, and its configuration was similar to a primary MH with no adherent cortical vitreous to its edges [77]. The other cases were characterized by “almost” FTMH with an overlying ILM drape [76,78,79]. Even in primary FTMH, release of vitreoretinal traction is not always necessary to close the MH [80]. In these cases, it is thought that the ELM and the outer nuclear layer served as a bridging element that facilitated reconstruction of the foveolar structures and MH closure [76,79]. Thus, the integrity of the ILM drape and ELM may distinguish a potentially reversible foveal defect from irreversible tissue loss. Chandra et al. [76] specifically suggested that an intact ILM drape, absence of vitreomacular traction and glial bridging may facilitate closure. These cases raise an important clinical question: Should every MacTel2-associated FTMH be operated on immediately? The literature does not currently provide a definitive answer.

MH secondary to MacTel2 is a relatively rare condition. There is not a single prospective randomized trial. The available evidence is currently limited to case reports, small retrospective series, and one relatively large multicenter retrospective study. Most of these reports suffer from a limited follow-up. All the reported cases are summarized in Table 1. The surgical repair of FTMH secondary to MacTel2 using standard PPV, posterior hyaloid removal and ILM peeling is usually less successful anatomically and less predictable functionally than the repair of a primary FTMH [81]. It has demonstrated limited success with closure rates ranging from 30 to 80% [82,83,84,85,86,87,88,89]. In 2009, Charbel-Issa and co-workers [82] were the first to attempt surgical correction with this standard technique in two eyes. In one eye the MH remained open and in the other eye, the MH size was decreased but remained open. Gregori and Flynn Jr [85] reported successful closure in one eye with improved visual acuity, and in another eye initial closure was achieved; however, the hole re-opened 4 months post-operatively. Based on these 2 cases they concluded that MH repair could be an effective treatment in these cases. Karth and colleagues [83] reported the outcomes of a small series of 4 eyes. In 2 eyes, surgery was unsuccessful. In another eye the MH closed but re-opened 4 months post-operatively. Only one eye achieved successful closure. In the largest study to date, 35% (12/34) of eyes closed, and in an additional 12% (4/34) of eyes, the MH was closed but an eccentric defect was present [84]. In contrast, Ahmad et al. [89] reported an 80% closure rate in 5 eyes. Re-opening of the MH has been reported in several cases [90,91].

ILM flap techniques, grafts, and newer adjuncts have improved closure rates over ILM peeling alone [83,84,92,93]. The largest case series reported a 91% (20/22 eyes) closure rate [84]. Despite the success reported with ILM flaps, Wang and colleagues [94] offer a cautionary tale. In their case report they noted that intraoperative indocyanine green staining revealed that the ILM was absent in parts of the macula which they attributed to Müller cell loss. Furthermore, they noted that initial closure was followed by late re-opening of the hole at 6 years post-operatively. In their view, which we also share, the current studies have a relatively short mean follow-up of 24 to 44 months [81,84,90]. An important question that has not been answered is whether the scaffolding function of the ILM flap diminishes with Müller cell function decline [94].

Free ILM patch grafts have also been used successfully in 3 eyes to close FTMHs secondary to MacTel2 [5].

In 2020 a patient with two prior unsuccessful surgical attempts to close a giant, chronic, 1355 μm FTMH underwent ART, perfluoro-octane tamponade for 1 week followed by silicone oil tamponade for 2 months. Subsequent PPV with a 20% sulphur hexafluoride (SF6) exchange achieved a successful closure. Remarkably his best corrected VA improved from logMAR 2 to logMAR 0.6. However it is unclear if the MH in this case was secondary to trauma or MacTel2 or both. Unfortunately there were no images to confirm the diagnosis of MacTel2. The pre-operative OCT is not typical of MacTel2 [95]. Park et al. [84] reported 5 ART procedures in their series. ART was performed as a primary procedure in one eye, as a secondary procedure after failed surgeries in 3 eyes, and as a repeat ART in 1 eye that suffered a dislocated ART graft. After a mean follow-up of 10 months, all eyes had closure of their MH [84].

The use of plasma rich in growth factors (PRGF) membrane as a biological adjuvant is promising. It was introduced in 2022 and has been used in 5 eyes [70,96,97]. The autologous PRGF membrane is obtained using the Endoret^®^ system (BTI Biotechnology Institute, Alava, Spain), where peripheral blood is drawn from the patient and centrifuged. The platelet fraction is obtained and activated with 10% calcium chloride and incubated at 37 °C for 30 min. The resulting membrane can then be grasped and placed over the MH. The main advantage of this technique is that ILM manipulation is not necessary [96]. Subsequently, Bertolani et al. [97] combined conventional ILM peeling with topical PRGF-Endoret^®^, also achieving complete anatomical closure while eliminating the need for postoperative face-down positioning.

Available observational evidence suggests a higher anatomical closure rate with inverted ILM flap techniques than with ILM peeling alone; however, comparisons are confounded by differences in hole size, disease severity, surgical selection, and follow-up, and functional superiority has not been established. Even though a recent narrative review of 95 eyes of 10 studies reported that both the inverted ILM flap and the ART techniques achieved the highest rates of closure when compared to standard PPV with posterior hyaloid removal and ILM peeling, these results were most likely driven by the outcomes of the largest series [84,92].

**Table 1:** Surgical outcomes of macular holes secondary to macular telangiectasia type 2.

Authors	N	Surgical Technique	Pre-Op VA (Snellen)	Lens Status	Anatomic Outcome	Post-Op VA (Snellen)	Follow-Up (Months)
Rishi and Kotari (2008) [86]	1	PPV ILM Peel	20/60	Pseudophakic	Open	20/100	3
Charbel Issa et al. (2009) [82]	2	PPV ILM Peel PPV ILM Peel and SO Tamponade	20/400 20/100	Not Specified Not Specified	Open Open	20/400 20/63	Not Specified Not Specified
Gregori & Flynn Jr (2010) [85]	2	PPV ILM Peel PPV ILM Peel	20/50 20/70	Phakic Phakic	Closed Closed then re-opened at month 4	20/30 20/70	Not Specified Not Specified
Sandhu and Steel (2010) [87]	2	PPV ERM Peel PPV ERM Peel	20/120 20/200	Pseudophakic Pseudophakic	Closed Closed	20/40 20/60	36 36
Shukla (2011) [88]	1	PPV ILM Peel	20/80	Not Specified	Closed	20/30	11
Karth et al. (2014) [83]	4	PPV ILM Peel PPV ILM Peel PPV ILM Peel PPV ILM Peel	20/100 20/100 20/100 20/200	Not Specified Not Specified Not Specified Not Specified	Open Closed then re-opened Open Closed	20/70 20/200 20/400 20/30	24 19 72 10
Lee et al. (2019) [98]	4	PPV ILM Peel PPV ILM Peel PPV ILM Peel PPV ILM Peel	20/126 20/40 20/200 20/200	Pseudophakic Phakic Pseudophakic Phakic	Closed Closed then re-opened at month 1 Closed Closed	20/32 20/100 20/32 20/400	43 6 24 10
Ahmad et al. (2020) [89]	5	PPV ILM Peel PPV ILM Peel PPV ILM Peel PPV ILM Peel PPV ILM Peel	20/80 20/80 20/50 20/200 20/60	2+ NS Cataract 2+ NS Cataract Phakic 2+ NS Cataract 2+ NS Cataract	Closed Closed Closed Closed Open	20/25 20/20 20/40 20/20 20/60	26 37 21 35 22
Miller et al. (2020) [90]	12	Not Specified	20/114	Not Specified	33.3% Closed	20/94	12
Hewson et al. (2020) [95]	1	PPV ART (Pre-Op OCT not typical for MacTel2 MH, 2 Prior Failed PPV ILM Peel)	20/2000	Pseudophakic	Closed	20/80	3
Knight et al. (2021) [99]	1	PPV Relaxing Retinotomy (Prior PPV ILM Peel)	20/200	Not Specified	Closed	20/100	6
Nishiyama et al. (2021) [91]	3	PPV ILM Flap PPV ILM Flap PPV ILM Flap	20/40 20/40 20/200	Not Specified Not Specified Not Specified	Closed Closed Closed then re-opened at month 6	20/20 20/25 20/60 to 20/100	24 24 24
Sborgia et al. (2021) [100]	1	PPV ILM Flap	20/40	Not Specified	Closed	20/20	3
Bozdogan et al. (2021) [93]	1	PPV Free ILM Graft (Prior PPV ILM Peel)	CF	Not Specified	Closed	20/100	2
Lee et al. (2024) [81]	17 9 1	PPV ILM Peel PPV ILM Flap PPV Free ILM Graft	20/83 (Did not report separately per procedure)	Not Specified	74.1% closed (Did not report separately per procedure)	20/51 (Did not report separately per procedure)	24 (Did not report separately per procedure)
de Oliveira Miranda et al. (2024) [101]	1	PPV Inverted ILM Flap	20/200	Not Specified	Closed	20/80	6
Park et al. (2024) [84]	34 22 5 1 1	PPV ILM Peel PPV ILM Flap PPV ART PPV Relaxing Retinotomy PPV Subretinal BSS Bleb	20/93 20/151 20/577 20/200 20/1400	19 Phakic 13 Phakic 1 Phakic 1 Phakic 0 Phakic	47% closed 91% closed 80% closed 100% closed 0% Closed	20/80 20/72 20/200 20/100 20/2000	30 18 10 48 36
Ozbek et al. (2025) [5]	6	PPV Free ILM Graft PPV ILM Flap PPV Free ILM Graft PPV ILM Peel PPV Free ILM Graft PPV ILM Flap	20/98 20/200 20/400 20/98 20/66 20/66	Not Specified Not Specified Not Specified Not Specified Not Specified Not Specified	Closed Closed Closed Closed Closed Closed	20/200 20/98 20/200 20/123 20/33 20/400	36 42 47 82 180 45
Usami et al. (2025) [102]	2	PPV ILM peel PPV Inverted ILM Flap	20/50 20/50	Pseudophakic Pseudophakic	Closed Closed	20/20 20/16	144 24
Wang et al. (2025) [94]	1	PPV Inverted ILM Flap	20/100	Not Specified	Closed then re-opened at 72 months	20/40 to 20/100	72
Rangel et al. (2022) [70]	1	PPV PRGF	20/200	Phakic	Closed	20/100	6
Bertolani et al. (2025) [97]	1	PPV ILM Peel PRGF	20/80	Phakic	Closed	20/25	12
Arias et al. (2026) [96]	3	PPV PRGF PPV PRGF (Prior PPV ILM Peel) PPV PRGF	20/100 CF 20/200	Not Specified Not Specified Not Specified	Closed Closed Closed	20/30 20/300 20/100	Median 6 (Not Specified for each individual case; 6 to 18)
Breazzano et al. (2026) [103]	3	PPV Amniotic Membrane Graft	20/150 20/400 20/125	Not Specified	Closed Closed Closed	20/70 20/80 20/125	6 10 24

VA = visual acuity; PPV = pars plana vitrectomy; ILM = internal limiting membrane; SO = silicone oil; ERM = epiretinal membrane; ART = autologous retinal transplant; OCT = optical coherence tomography; MH = macular hole; PRGF = plasma rich in growth factors.

### 6.4. Long-Term Anatomical Stability and Macular Hole Re-Opening

A key distinction between MacTel2-associated and primary MH is that surgical closure does not arrest the underlying retinal degeneration. Müller cell dysfunction, foveal cavitation, photoreceptor loss, and outer retinal atrophy may continue after successful repair, allowing visual decline to occur independently of the anatomical status of the MH. Long-term prognosis therefore reflects both the initial success of surgical reconstruction and the subsequent trajectory of MacTel2-related neurodegeneration.

Reopening of an initially closed FTMH is an uncommon but well-documented complication [83,84,85,90,91,94]. Unlike recurrence of a primary MH, which is often related to persistent or recurrent vitreofoveal traction, reopening in MacTel2 appears more plausibly attributable to progressive retinal tissue loss and to weakening of the foveal architecture. Müller cells are particularly relevant because of their structural role within the fovea. Continued dysfunction or loss may permit recurrent cavitation and tissue collapse, potentially overcoming the structural support provided by an ILM flap or other reconstructive technique. Larger preoperative cavitations and more advanced outer retinal atrophy have also been associated with unfavorable outcomes, although these observations are derived primarily from small series and individual cases [83,84,85,90,91,94].

The possibility of very late recurrence is illustrated by a 2025 report describing reopening 6 years after initially successful ILM flap surgery [94]. Patchy ILM deficiency was identified intraoperatively, while three-dimensional swept-source OCT angiography demonstrated progressive structural and vascular collapse. Although the hole initially closed with visual improvement, it subsequently reopened with corresponding visual decline. This observation suggests that flap-based surgery can restore foveal continuity and provide structural reinforcement but cannot prevent continued degeneration of the surrounding retina. Long-term anatomical stability may therefore depend more on the rate of MacTel2 progression than on the technical success of the original repair.

The available literature is insufficient to establish a reliable recurrence rate or to quantify the contribution of individual risk factors. Nevertheless, documented late reopening, including recurrence several years after apparently successful surgery, supports prolonged postoperative surveillance. Patients should be counseled that surgery addresses the mechanical complication of MH but does not modify the underlying neurodegenerative process. Accordingly, anatomical closure represents an important therapeutic endpoint, but preservation of viable outer retinal tissue and the subsequent course of MacTel2 are likely to be more important determinants of sustained visual function [83,84,94].

### 6.5. Functional Outcomes

Published evidence regarding the functional outcomes of MH secondary to MacTel2 remains limited to case reports, small retrospective series, comparative observational studies, and one large multicentre study. Collectively, these studies indicate that visual recovery is influenced not only by anatomical closure of the hole but also by the integrity of the underlying foveal tissue. In particular, pre-existing Müller cell dysfunction, photoreceptor loss, outer retinal atrophy, and progressive neurodegeneration may limit visual improvement despite successful anatomical repair [81,90,92,100,104]. Accordingly, anatomical success should not be equated with functional recovery. Visual outcomes are also potentially confounded by subsequent cataract surgery and continued progression of MacTel2.

Early reports of surgery for MacTel2-associated FTMH generally described less favorable anatomical and functional outcomes than those expected for primary MH. Miller and associates [90] compared 12 surgically treated eyes with 26 eyes managed conservatively and found no statistically significant change in VA from baseline at either 3 or 12 months in the surgical group. In contrast, the largest multicenter study of 50 eyes reported improvement of at least two lines in 57% of eyes overall, with better functional outcomes observed after ILM flap surgery than after ILM peeling. Subjective improvement in central scotomas was reported in 80% of eyes [84]. Conversely, a more recent Turkish series reported postoperative VA loss from baseline despite anatomical closure [5]. Taken together, these findings emphasize that the visual response to surgery is heterogeneous and cannot be predicted solely from whether the hole closes.

Conventional PPV with ILM peeling frequently failed to achieve closure, and visual improvement remained limited even when anatomical repair was successful [83,92]. This may reflect the fact that foveal function is already compromised by MacTel2 before development of a full-thickness defect. In the series of Karth et al. [83], only one of four eyes treated with PPV, ILM peeling, gas tamponade, and postoperative prone positioning remained closed, with a final visual acuity (VA) of 20/30. Two eyes failed to close and achieved final VAs of 20/70 and 20/200, while another reopened after 4 months and ultimately reached 20/400. Notably, the successfully repaired eye had relatively limited preoperative cavitation, whereas the eyes with unfavorable outcomes demonstrated more extensive cavitary changes. These observations support the concept that the extent of pre-existing retinal tissue loss may be more important for prognosis than vitreomacular traction alone [83]. Subsequent reports demonstrated that meaningful visual recovery is possible in appropriately selected eyes. Ahmad et al. [89] reported closure in four of five eyes undergoing PPV, ILM peeling, and gas tamponade, with final VAs ranging from 20/20 to 20/40 after more than 20 months of follow-up. The single eye that remained open achieved 20/60. A larger study further demonstrated the variable functional response to surgery [84]. Among eyes undergoing ILM peeling with successful closure, mean VA changed from 0.667 logMAR preoperatively to 0.602 logMAR at 6 months, without a statistically significant difference. Nevertheless, 43% of eyes gained at least two lines and 24% gained at least four lines [84]. Although these findings indicate that conventional surgery can produce substantial visual improvement, their interpretation is limited by the small sample size, lack of a control group, and relatively favorable preoperative VA. More broadly, the disparate results of these early series likely reflect the structural heterogeneity of MacTel2-associated FTMH. Eyes with a relatively focal defect and preserved surrounding foveal tissue may retain considerable visual potential, whereas holes developing in the setting of extensive cavitation and outer retinal degeneration are less likely to regain useful vision. These findings illustrate that population-level mean VA may obscure clinically meaningful improvement in individual eyes while simultaneously indicating that conventional ILM peeling does not consistently restore vision.

Outcomes appear more favorable with ILM flap techniques [84,92]. Nishiyama and Iwase [91] reported three patients treated with PPV, inverted ILM flap placement, gas tamponade, and postoperative prone positioning. All three holes initially closed. Two remained closed for at least 2 years, with final VAs of 20/20 and 20/25. In the third eye, the hole reopened at 6 months; VA had improved to 20/60 before reopening but subsequently declined to 20/100. Park et al. [84] reported that at 6 months postoperatively, the mean VA improved significantly to 0.555 logMAR (~20/70) from 0.878 logMAR (~20/150) at baseline, with 56% of patients gaining at least 2 lines of vision, and 28% gaining 4 or more lines [84]. These results demonstrate both the potential for substantial visual recovery following flap-based repair in selected eyes and the possibility that successful initial closure may not provide permanent structural stability [91,92]. However, these results cannot establish the superiority of ILM flap surgery because the comparison was observational and the treatment groups differed in baseline characteristics and hole dimensions. The findings nevertheless suggest that techniques providing additional structural support may be advantageous in appropriately selected MacTel2-associated FTMHs.

ART has primarily been used for large, chronic, or refractory MHs and therefore has generally been performed in eyes with advanced disease and poor baseline vision [84,92,95]. In Park and co-workers’ [84] study, the mean preoperative VA of eyes undergoing ART was 1.460 logMAR (~20/600). Although all five eyes achieved anatomical closure, the mean VA at 6 months improved to 1.000 logMAR (~20/200), a change that did not reach statistical significance; only 25% of eyes gained at least two lines [84]. Exceptional visual improvement has nevertheless been reported. Hewson et al. [95] described closure of a giant 1355 µm MH accompanied by improvement in VA from counting fingers to 20/80 at 3 months. Visual field testing also demonstrated recovery of macular function in three quadrants. Again the lack of images precludes diagnosing the exact etiology of the MH (trauma vs. MacTel2) [95]. These findings suggest that ART may be effective for achieving anatomical closure in otherwise difficult cases, but functional recovery remains limited by the underlying retinal degeneration.

Biologic adjuncts have also been explored as strategies to facilitate closure while minimizing manipulation of the already compromised fovea. In one case treated with a PRGF membrane, anatomical closure was achieved but VA remained unchanged at 20/200 at 6 months [70]. Others have reported four additional cases where all eyes exhibited various degrees of visual improvement [96,97]. These isolated observations suggest potential value for biologic adjuncts, but current evidence is insufficient to determine their independent contribution to visual recovery.

The functional outcome of MH surgery in MacTel2 differs fundamentally from that of primary FTMH. Contemporary techniques, particularly inverted ILM flap procedures, appear to improve anatomical closure and may provide greater functional benefit than conventional ILM peeling; however, visual recovery remains highly variable. The available evidence indicates that the condition of the surrounding retina, particularly EZ area, EZ/ELM integrity, extent of intraretinal cavitation, photoreceptor preservation, and outer retinal atrophy, is likely to be more informative for functional prognosis than MH diameter alone. The long-term bilateral case reported by Usami et al. [102] illustrates the importance of assessing retinal function beyond conventional visual acuity. Despite postoperative visual acuities of 20/32 at 1 year and 20/20 at 12 years in one eye and 20/16 at 2 years in the fellow eye, microperimetry demonstrated areas of reduced retinal sensitivity corresponding to regions of retinal atrophy. These findings suggest that visual acuity may overestimate functional recovery by failing to capture localized retinal dysfunction.

## 7. Future Directions

The rarity of MH secondary to MacTel2 has precluded prospective randomized trials, and the available evidence remains largely limited to case reports, small retrospective series, and one relatively large multicenter retrospective study, often with limited follow-up. Substantial heterogeneity exists in surgical techniques, definitions and classifications of MH, OCT parameters, MacTel2 staging, reporting of EZ area and ELM integrity, and in duration of follow-up. Functional outcomes have also been assessed inconsistently. The absence of randomized comparative studies, together with small sample sizes, short follow-up in many reports, and potential publication bias toward anatomically successful cases, limits the strength and generalizability of the available evidence. Consequently, important questions regarding pathogenesis, prognosis, and optimal management remain unresolved.

The relative contributions of Müller cell dysfunction, intraretinal cavitation, progressive foveal tissue loss, outer retinal degeneration, and vitreomacular traction to MH formation are incompletely understood. In addition, there are no validated biomarkers to identify eyes at increased risk of developing a FTMH or to predict functional recovery after surgical repair. Candidate OCT biomarkers—including minimum hole diameter, residual foveal tissue thickness, extent of intraretinal cavitation, EZ area, ELM integrity, and the extent of surrounding outer retinal atrophy—may provide prognostic information but have not been systematically validated. Importantly, anatomical closure does not necessarily translate into meaningful visual recovery, underscoring the need for functional outcomes beyond visual acuity, such as microperimetry, retinal sensitivity, and patient-reported visual function. Prospective longitudinal studies integrating multimodal imaging, including OCT, OCT angiography, fundus autofluorescence, and adaptive optics, are needed to identify predictors of surgical benefit, clarify the relationship between anatomical closure and functional recovery, and distinguish visual dysfunction attributable to the MH from irreversible MacTel2-related neurodegeneration.

The optimal surgical strategy also remains uncertain. Although inverted ILM flap techniques have been associated with higher closure rates and potentially greater visual improvement than conventional ILM peeling, the available comparative evidence is limited and does not establish definitive superiority. The roles of alternative approaches, including ILM grafts, autologous retinal transplantation, and biologic adjuncts such as PRGF, remain investigational. The biological basis for the apparent benefit of scaffold-based techniques warrants further study, particularly given the central role of Müller cell degeneration in MacTel2. It remains unclear whether an ILM flap provides primarily mechanical support or may also facilitate Müller cell migration, glial remodeling, or restoration of foveal architecture. Similarly, there is currently no standardized classification of MH severity within the MacTel2 disease spectrum, limiting comparisons among studies and the development of stage-specific treatment strategies.

An additional unmet need is the prevention of late MH reopening. Although uncommon, reopening has been reported after initially successful repair and may reflect continued Müller cell dysfunction, progressive cavitation, foveal tissue loss, and outer retinal degeneration rather than recurrent vitreofoveal traction alone. Long-term multimodal imaging may help determine whether anatomical closure alters the trajectory of functional decline or merely addresses the structural manifestation of an ongoing neurodegenerative process. This distinction is particularly important when evaluating emerging treatments. It remains unknown whether combining surgical repair with disease-modifying therapy, including approaches targeting Müller cells or retinal neurodegeneration such as CNTF, could improve the durability of closure or preserve visual function. Ultimately, surgery addresses the structural complication but does not necessarily modify the underlying disease process. Future research should therefore extend beyond strategies designed solely to achieve anatomical closure toward interventions that preserve or restore viable Müller cell, photoreceptor, and outer retinal architecture, prevent MH formation, and maintain long-term foveal function. Disease-modifying and regenerative approaches may ultimately be required to improve both the durability of anatomical repair and functional outcomes in MacTel2-associated MH.

## Figures and Tables

**Figure 1:**
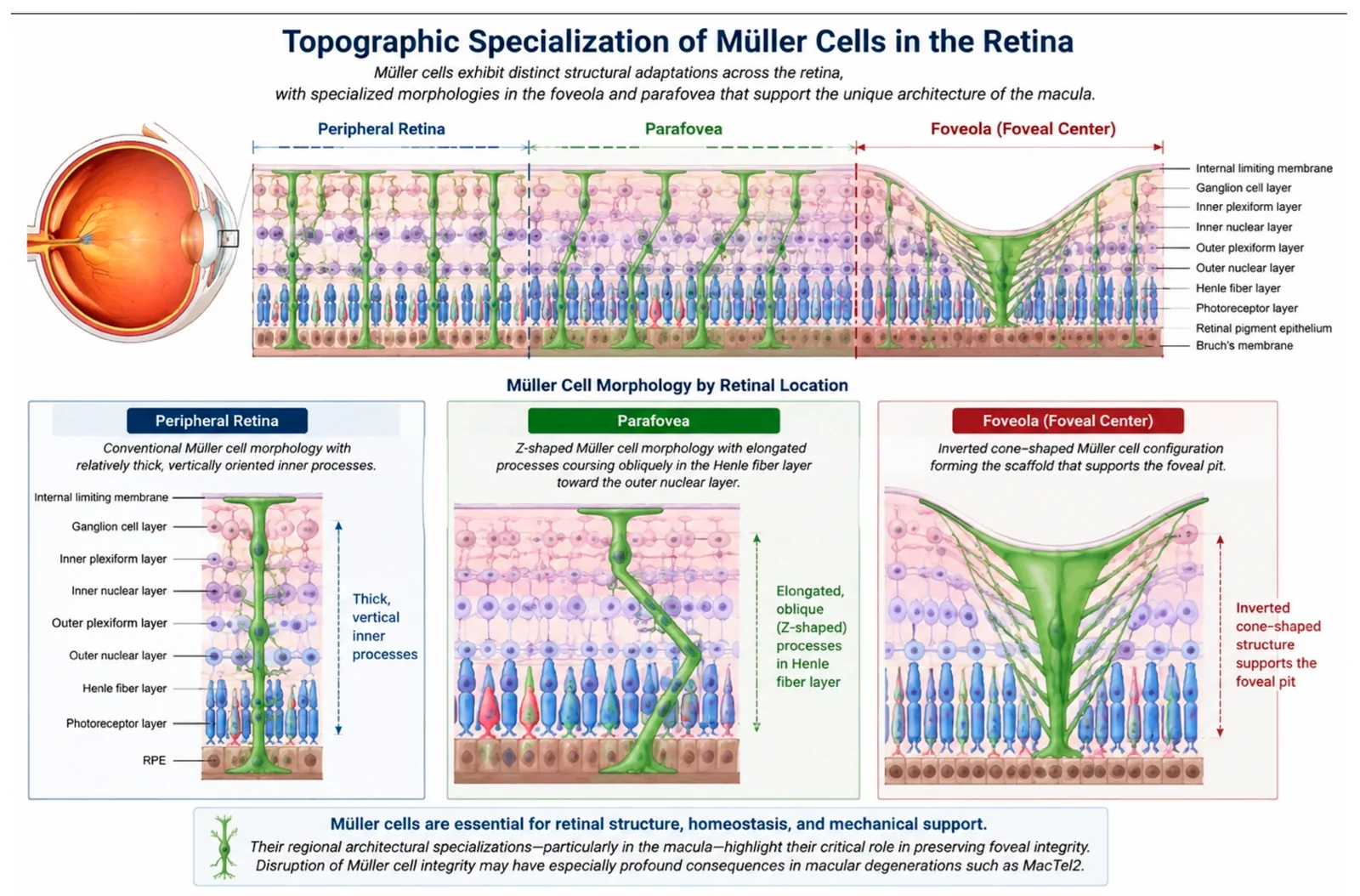
Topographic Specialization of Müller Cells in the Retina.

**Figure 2:**
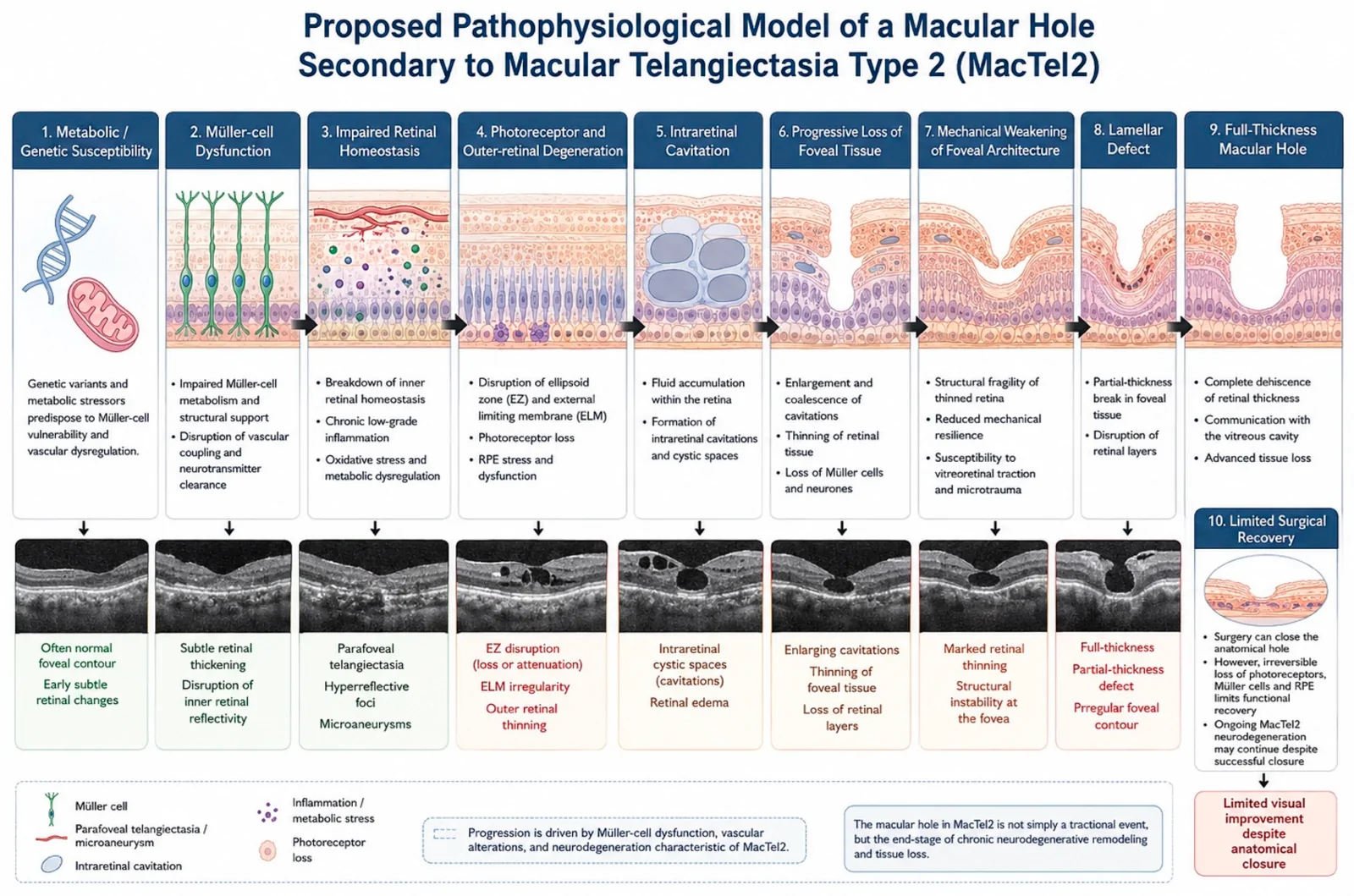
Proposed Pathophysiological Model of a Macular Hole Secondary to Macular Telangiectasia Type 2 (MacTel2).

**Figure 3:**
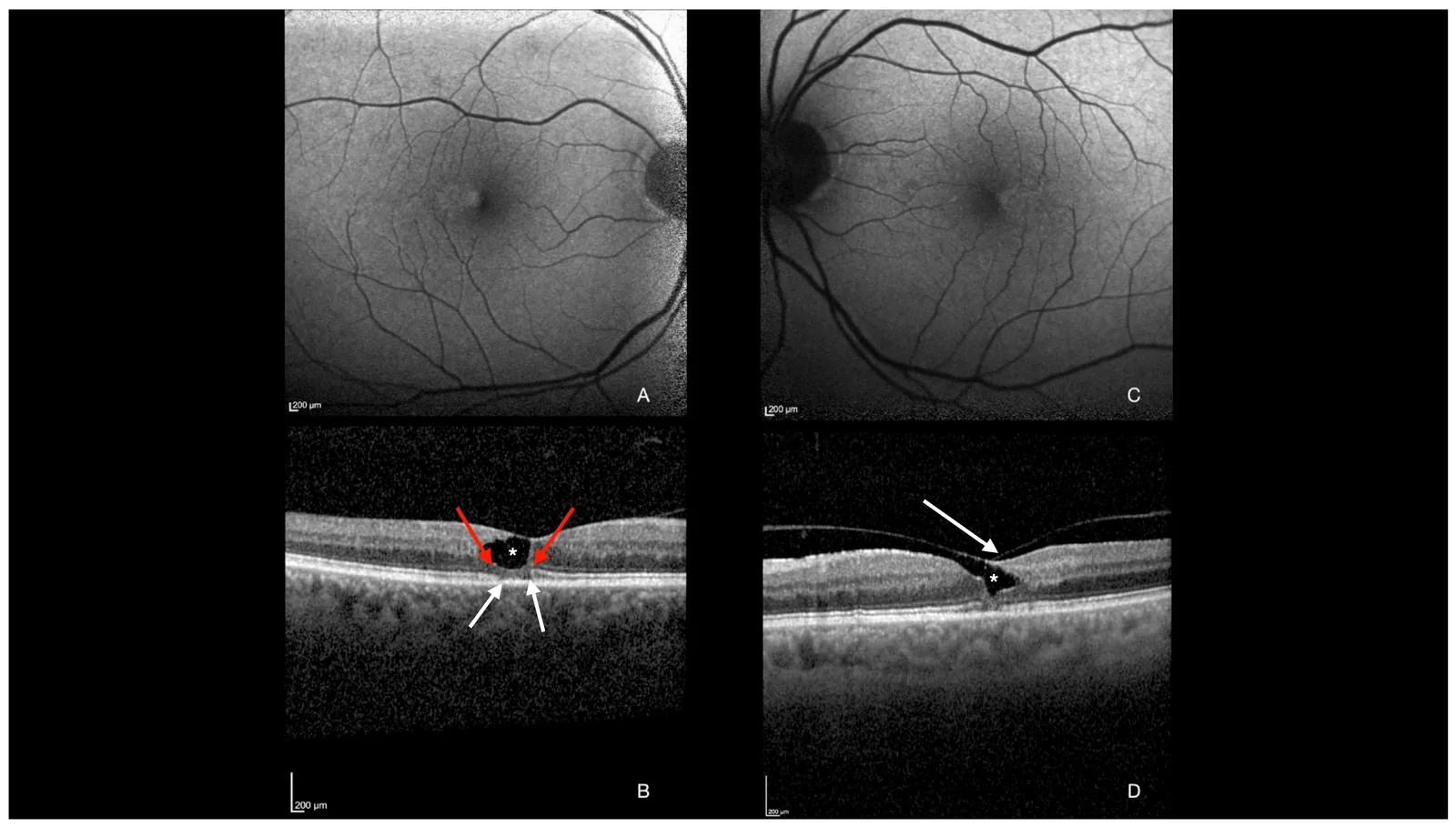
A case where vitreofoveal traction contributes to the formation of a LMH. (**A**) Focal area of increased FAF in the temporal parafovea; (**B**) SD-OCT showing an inner retinal cavitation with discontinuity of the ELM and EZ bands; (**C**) Focal area of increased FAF in the temporal parafovea; (**D**) SD-OCT showing vitreofoveal traction creating a LMH.

**Figure 4:**
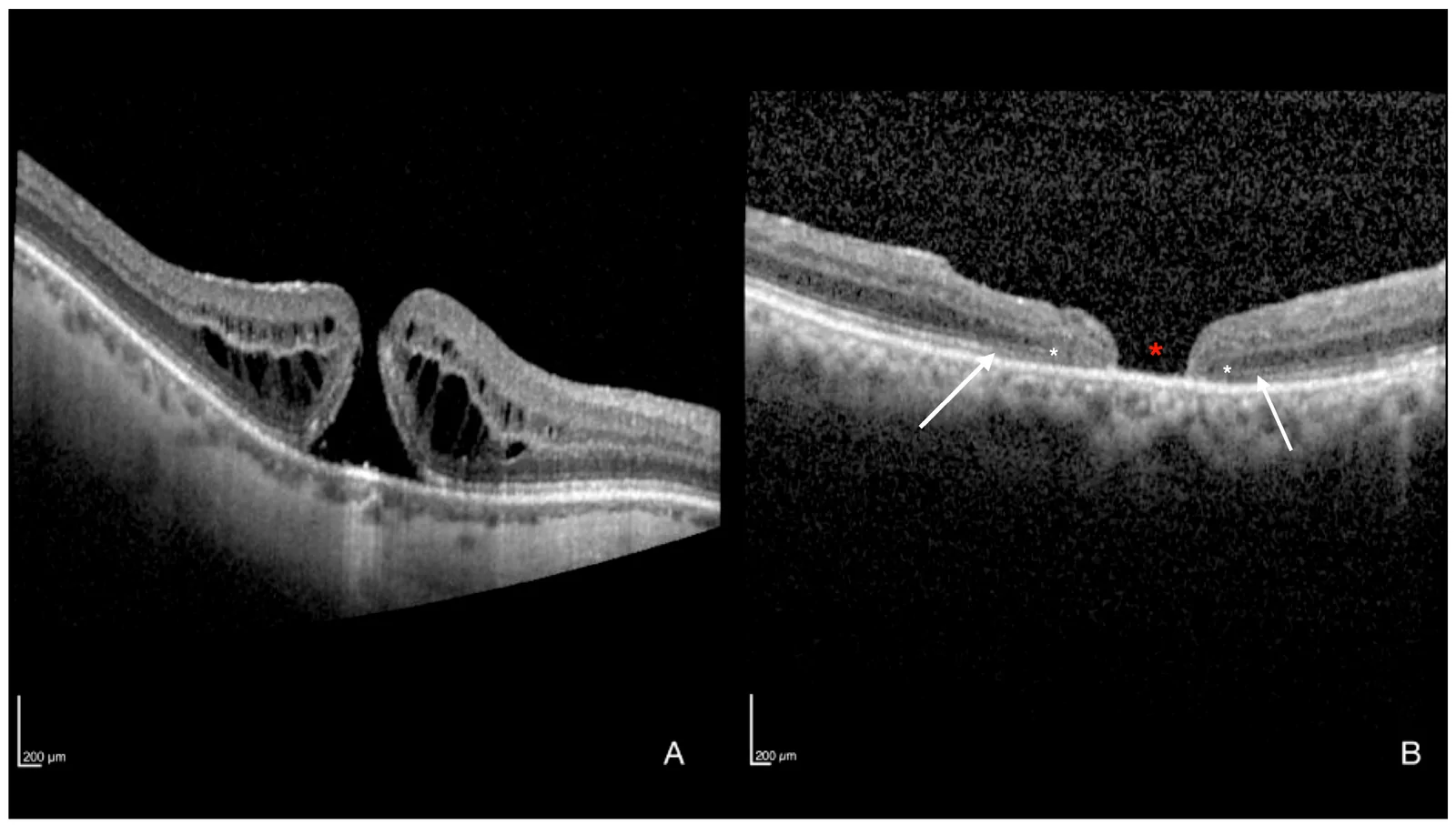
Differences between primary FTMH and FTMH Secondary MacTel2. (**A**) SD-OCT of a primary FTMH; (**B**) SD-OCT of a FTMH secondary to MacTel2. Note the disrupted EZ and ELM on the temporal side of the MH. The inner retina is also thinner on the temporal side.

## Data Availability

No new data were created or analyzed in this study. Data sharing is not applicable to this article.
